# ICU patients treated with RRT for AKI who have chronic kidney disease have better 1-year outcome compared with patients with better kidney function

**DOI:** 10.1186/cc13595

**Published:** 2014-03-17

**Authors:** W De Corte, V Sergoyne, J Vanhalst, A Dhondt, S Claus, S Oeyen, J Decruyenaere, E Hoste

**Affiliations:** 1 AZ Groeninge, Kortrijk, Belgium; 2 Stedelijk Ziekenhuis, Aalst, Belgium; 3 Sint- Jozefziekenhuis, Izegem, Belgium; 4 Ghent University Hospital, Ghent, Belgium

## Introduction

Chronic kidney disease (CKD) is a risk factor for developing acute kidney injury (AKI) with need for renal replacement therapy (RRT). AKI-RRT is associated with important short-term mortality, and recent data showed there is also important increased risk for 1-year mortality. The aim of this study is to evaluate variables associated with 1-year survival, and in particular the impact of baseline CKD in a cohort of AKI-RRT patients.

## Methods

A single-center observational study in a 50-bed ICU tertiary care hospital. During the study period August 2004 to December 2012 all consecutive adult AKI-RrT patients were included. Data were retrieved from the electronic ICU patient file, the electronic hospital patient file and the electronic ICU-RRT database. Long-term outcome data were collected by a telephone survey.

## Results

During the 9-year study period, a total of 1,291 AKI-RRT patients were included. Short-term mortality at day 30 was 47.2%; mortality at 1 year was 64.3%. Compared with nonsurvivors, 1-year survivors had similar age (65 vs. 67 years, *P *= 0.077), worse kidney function at baseline (eGFR 46 vs. 52 ml/minute/1.73 m^2^, *P *= 0.001; CKD stage ≥3 65% vs. 58%, *P *= 0.019), a greater proportion was male (69.0% vs. 63.2%, *P *= 0.048), and more were admitted to the cardiac surgery ICU (39% vs. 46%, *P *= 0.012). They were less severely ill as illustrated by lower SAPS 2 score at ICU admission (52 vs. 69, *P *< 0.001), and at the time of initiation of RRT, and a lower SOFA score (9 vs. 11; *P *< 0.001). A smaller proportion was on mechanical ventilation (85.1% vs. 89.7%, *P *= 0.042) and on vasoactive drugs (45.7% vs. 63.9%, *P *< 0.001). Survivors had earlier initiation of RRT (2 vs. 3 days, *P *= 0.034), and more frequently intermittent RRT was used (84.8% vs. 63.2%; *P *< 0.001). When corrected for gender, age, severity of illness, and modality and timing of RRT, worse baseline kidney function, defined as CKD stage ≥3, remained associated with better 1-year survival (odds ratio 1.6, 95% CI: 1.1 to 2.2, *P *= 0.011).

## Conclusion

In ICU patients who had AKI-RRT, 1-year survival was associated with lower severity of illness. Surprisingly, worse kidney function or CKD stage ≥3 was also associated with long-term survival. This effect remained present after adjudication for relevant covariates.

